# Radiogenomic modeling of EGFR mutation status in brain metastases from lung adenocarcinoma: a multicenter study with biological interpretability

**DOI:** 10.1038/s41746-026-02931-9

**Published:** 2026-07-06

**Authors:** Fuxing Deng, Xianjing Chu, Wen Shi, Gang Xiao, Guilong Tanzhu, Lishui Niu, Zijian Zhang, Rongrong Zhou, Guang Yang

**Affiliations:** 1 The department of oncology, Xiangya Hospital, Central South University, Changsha, China; 2 National Clinical Research Center for Geriatric Disorders, Xiangya Hospital, Central South University, Changsha, China; 3 Xiangya Lung Cancer Center, Xiangya Hospital, Central South University, Changsha, China; 4 Bioengineering Department and Imperial-X, Imperial College London, London, UK; 5 National Heart and Lung Institute, Imperial College London, London, UK; 6 Cardiovascular Research Centre, Royal Brompton Hospital, London, UK; 7 School of Biomedical Engineering & Imaging Sciences, King’s College London, London, UK

**Keywords:** Biomarkers, Cancer, Computational biology and bioinformatics, Genetics, Oncology

## Abstract

Accurate prediction of epidermal growth factor receptor (EGFR) mutation status in lung adenocarcinoma (LUAD) with brain metastases (BMs) is crucial for guiding targeted therapy. However, noninvasive and biologically interpretable tools remain limited. In this multicenter radiogenomic study, we analyzed a total of 1303 BMs from 421 LUAD patients across three institutions. 3435 radiomic features were extracted from T1, T2, and contrast-enhanced T1 sequences. A four-task classification framework was developed to predict EGFR mutation status (EGFR+, 19Del, L858R, or sensitizing mutation) using an adaptive LightGBM-based modeling pipeline. The models achieved excellent performance in the internal cohort (AUCs up to 0.95) and were further validated in 94 lesions with pathologically confirmed EGFR status, reaching an accuracy of 83.0%, sensitivity of 84.7%, and specificity of 80.0%. SHAP and LIME analyses revealed that shape-based radiomic features, particularly original_shape_sphericity, were the most important predictors of EGFR mutational subtypes. Then, we conducted transcriptomic analysis on 38 matched surgical specimens. Radiogenomic correlation revealed that sphericity negatively correlated with RNF125 and SLC37A2. Downstream enrichment analysis identified EGFR-associated features linked to DNA replication, sister chromatid segregation, and ERBB signaling. The study demonstrates that radiogenomic modeling, grounded in interpretable biology, holds promise as a non-invasive, clinical strategy for precision stratification of LUAD BMs.

## Introduction

Lung cancer remains the leading cause of cancer-related mortality globally^1^. It is primarily classified into non-small cell lung cancer (NSCLC) and small cell lung cancer (SCLC)^2^. Among NSCLC subtypes, lung adenocarcinoma (LUAD) is the most prevalent, accounting for approximately 40% to 50% of all lung cancer cases. One of the most formidable clinical challenges in NSCLC management is the development of brain metastases (BMs), which occur in nearly half of all patients throughout the disease course^3^. BMs significantly contribute to mortality, and the blood-brain barrier (BBB) limits the efficacy of conventional chemotherapeutic agents^4^. The advent of epidermal growth factor receptor (EGFR)-targeted therapies has transformed the therapeutic landscape for LUAD, particularly for patients with BMs, by markedly improving survival in those harboring actionable EGFR mutations^5^. Consequently, accurate identification of EGFR mutation status is essential to guide precision treatment strategies.

Currently, pathological biopsy remains the gold standard for EGFR mutation detection^6^. However, obtaining tissue samples from BMs is often invasive and technically challenging, with potential procedural risks^7,8^. Although liquid biopsy approaches such as circulating tumor DNA (ctDNA) analysis show promise, they are constrained by low sensitivity and high cost, limiting widespread clinical utility^9^. Therefore, there is an urgent need for non-invasive, cost-effective alternatives for molecular diagnosis. Radiomics, a quantitative extraction of high-dimensional data from standard medical images using advanced computational algorithms, has emerged as a powerful, non-invasive tool that captures comprehensive tumor characteristics while minimizing sampling bias^10^. Our prior studies have demonstrated that radiomics-based approaches can effectively differentiate gliomas from BMs and distinguish BMs arising from LUAD versus squamous cell carcinoma^11,12^. These findings highlight the ability of imaging phenotypes to reflect underlying tumor biology and histological origin. However, substantial biological heterogeneity exists among patients with different molecular subtypes, and imaging features alone are insufficient to fully capture the complex molecular landscape of tumors. In this context, radiogenomics, an emerging field that integrates radiomic features with genomic data, has garnered increasing attention in recent years. This integrative approach facilitates a shift from purely anatomical imaging toward molecular-level characterization, enabling the use of radiomic features as surrogate biomarkers for tumor genotyping and ultimately advancing precision oncology.

However, most existing radiomics-based EGFR mutation predictions have been limited by single-center designs, lack of external validations, and reliance on primary tumors rather than direct validation from BMs. Furthermore, the biological mechanisms underlying critical radiomic features have rarely been elucidated, significantly restricting their clinical interpretability and application^13–15^. Notably, comprehensive radiogenomic studies centered on magnetic resonance imaging (MRI)-derived features from LUAD BMs are exceedingly rare, highlighting a critical gap in understanding the underlying biological mechanisms that drive these imaging phenotypes.

To address these critical gaps, this retrospective study aims to: (1) develop and externally validate a radiomics-based model from MRI images to accurately predict EGFR mutation status directly from BMs in LUAD patients; (2) validate radiomic predictions using matched BMs’ specimens; and (3) explore the biological mechanisms associated with significant radiomic features using RNA sequencing, thereby advancing radiogenomics and precision oncology.

## Results

### Patient characteristics

**Fig. 1:**
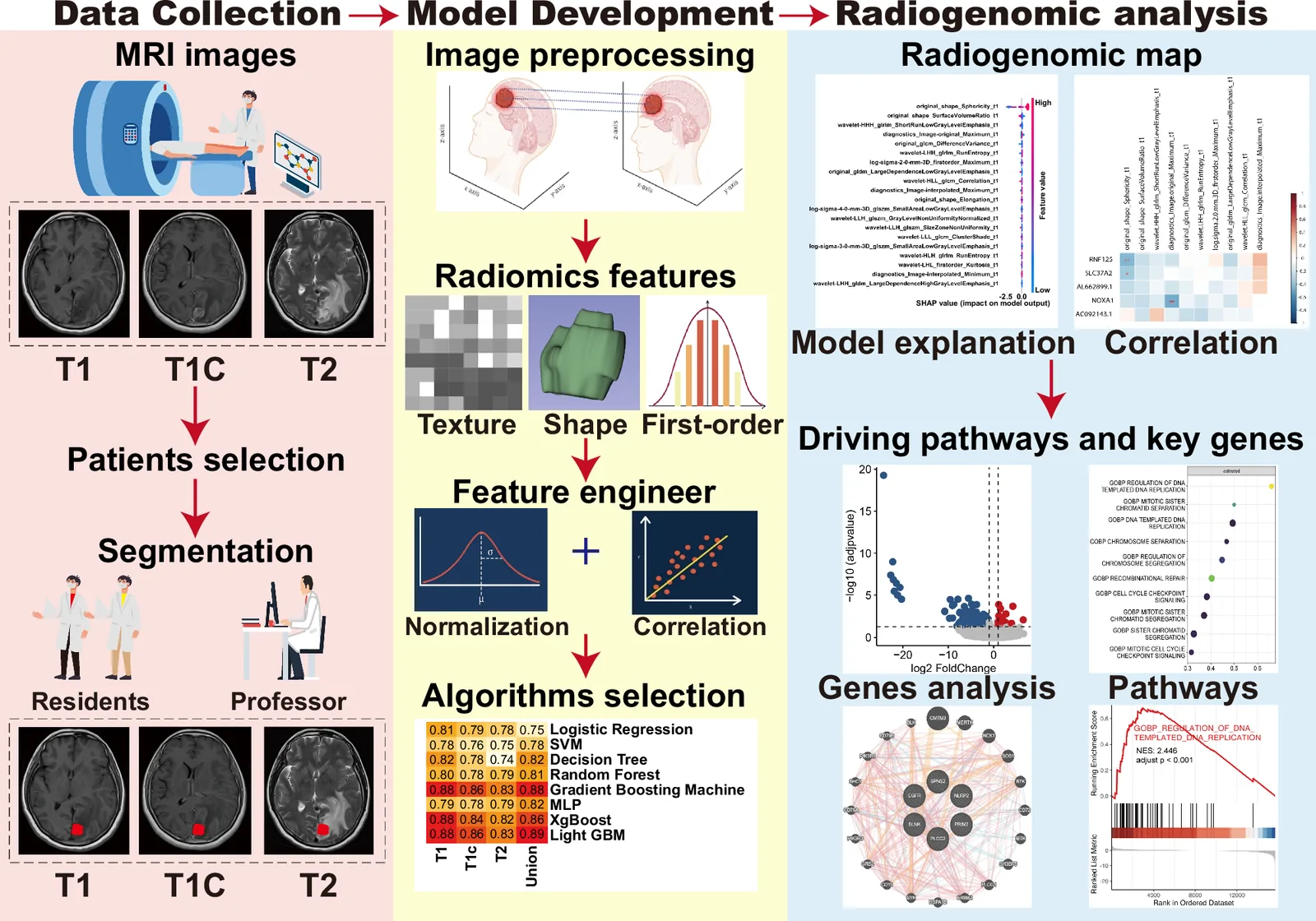
Workflow of the study. Patients were screened and enrolled based on inclusion and exclusion criteria. Regions of interest (ROIs) were delineated on the acquired multimodal MRIs, from which radiomic features were subsequently extracted. These features were categorized into first-order, second-order, and higher-order groups. Extracted features underwent further processing. Eight algorithms were deployed for modeling. The one with the highest AUROC underwent hyperparameter optimization. The model interpretation was achieved by calculating the SHAP values. The most contributory radiomic features were grouped into high and low-expression categories. Following patient grouping, differences in genomic expression were analyzed, and enriched pathways were identified.

A total of 421 patients with BMs from LUAD were included in this study, encompassing 1303 brain lesions, 1088 from the internal cohort, 121 from the external validation cohort, and 94 from the tissue validation cohort. Detailed demographic and clinical characteristics of both cohorts are summarized in Table 1. Gender distribution was similar among cohorts, with males comprising 49.2% in the internal cohort, 47.5% in the tissue validation cohort, and 59.6% in the external validation cohort. The average age across their cohorts were 57 ± 6 years, 57 ± 6 years, and 65 ± 9 years. The internal cohort included 225 EGFR-positive and 96 EGFR-negative patients, while the external validation cohort had a relatively higher proportion of EGFR-negative cases (44.7%). Among EGFR-positive patients in the internal cohort, 97 harbored exon 19 deletions (19Del), 95 carried L858R mutations, and 33 presented with other non-sensitive EGFR mutations such as exon 20 insertions and exon 18 deletions. An overview of the study design is summarized in Fig. 1.

**Table 1:** Baseline characteristics of enrolled patients

Variables	Internal cohort (*N* = 321)	Tissue validation cohort (*N* = 53)	External validation cohort (*N* = 47)
Age, Mean ± SD	57 ± 6	57 ± 6	65 ± 9
Gender, *n* (%)			
Male	158 (49.2)	27 (50.9)	28 (59.6)
Female	163 (50.8)	26 (49.1)	19 (40.4)
KPS, Median (Q1,Q3)	95 (90, 100)	95 (90, 100)	95 (90, 100)
EGFR status (%)			
EGFR^wt^	96 (29.9)	27 (50.9)	21 (44.7)
EGFR^+^	225 (70.1)	26 (49.1)	26 (55.3)

### Model development and performance evaluation

A total of 3264 regions of interest (ROIs) were delineated from MRI images of the internal cohort, with 1145 radiomic features extracted per ROI using PyRadiomics (Supplementary Table 1). To optimize classification performance, 384 models were trained and tested across 8 machine learning algorithms, 3 feature engineering strategies, 4 classification tasks (Task 1–4), and 4 sequence combinations (T1, T2, T1C, and multimodal fusion). Among all models, LightGBM consistently demonstrated superior performance, especially when combined with Spearman correlation filtering and *Z*-score normalization (Supplementary Fig. 1 and Supplementary Table 2).

Two fusion strategies were evaluated. Interestingly, models trained on single-sequence T1 images performed comparably, and in some cases better than those using multimodal feature fusion, particularly in predicting EGFR mutation status (Supplementary Fig. 1). Given the principle of model parsimony (Occam’s razor), we selected the optimal single sequence for each classification task to balance model complexity with performance. T1WI achieved the highest accuracy in Tasks 1–3, while T2WI outperformed others in Task 4.

**Fig. 2:**
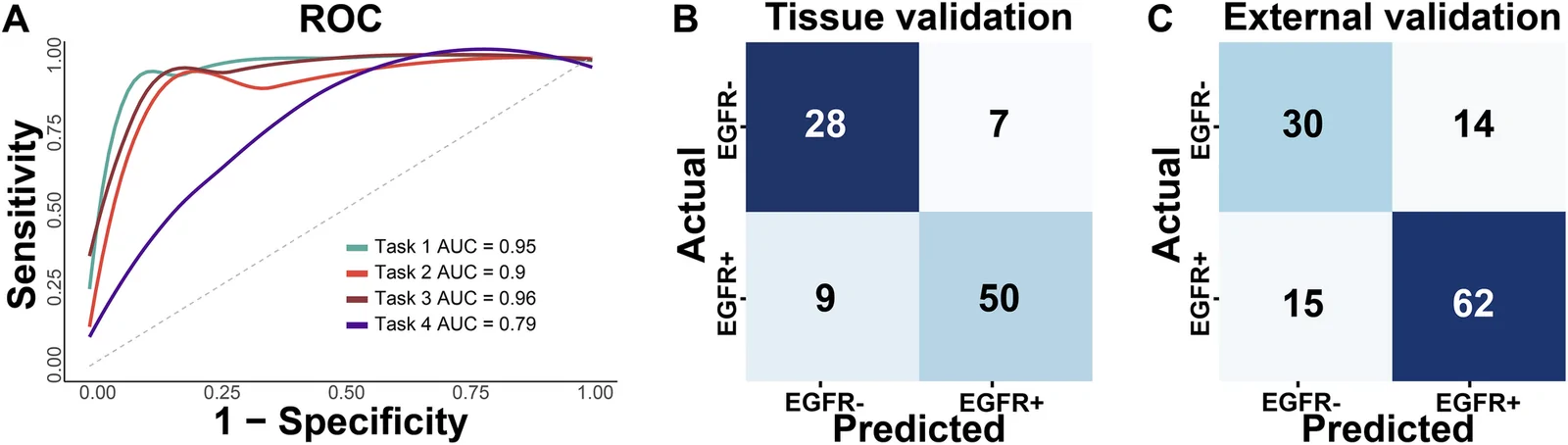
Performance of optimized radiogenomic models. **A** ROC Curve plot generated following hyperparameter optimization. All predicting mutation status models were optimized using LightGBM. Task 1: To determine the presence of any EGFR mutation in patients. Task 2: To identify patients with the Del19 mutation. Task 3: To identify patients with the L858R mutation. Task 4: To determine whether patients have either the Del19 or L858R mutation. **B** Confusion matrix of the optimal model in the tissue validation cohort. **C** Confusion matrix of the optimal model in the external validation cohort.

After hyperparameter tuning using an adaptive optimization training pipeline (AOTP), final AUROC scores in the test cohort were as follows (Fig. 2A): Task 1 (EGFR vs. wild-type): AUROC = 0.95 (95% CI: 0.93–0.96). Task 2 (19 Del): AUROC = 0.92 (95% CI: 0.91–0.93). Task 3 (L858R): AUROC = 0.92 (95% CI: 0.91–0.93). Task 4 (19 Del or L858R): AUROC = 0.79 (95% CI: 0.71–0.87).

### External validation and tissue-level confirmation

To validate the model’s generalizability and clinical applicability, we applied the optimal LightGBM model to the tissue validation cohort comprising 94 BMs with confirmed EGFR mutation status from surgical specimens. As shown in Fig. 2B, the model achieved an accuracy of 83.0%, with a sensitivity of 84.7% and a specificity of 80.0%. These results affirm the model’s robustness and potential utility as a non-invasive diagnostic tool in clinical practice.

As shown in the confusion matrix (Fig. 2C), among the 121 BMs in the external validation, the model correctly classified 62 EGFR-mutant lesions (true positives) and 30 wild-type lesions (true negatives), while misclassifying 15 mutant lesions as wild-type (false negatives) and 14 wild-type lesions as mutant (false positives). Correspondingly, the model achieved an accuracy of 76.0% (95%CI: 67.7%–82.8%), a sensitivity of 80.5% (95%CI: 70.3%–87.8%), and a specificity of 68.2% (53.4%–80.0%) in this independent cohort. These results demonstrate the model’s robust generalizability across different clinical centers and scanner parameters, further supporting its potential for real-world clinical deployment.

### Model interpretability using SHAP and LIME

**Fig. 3:**
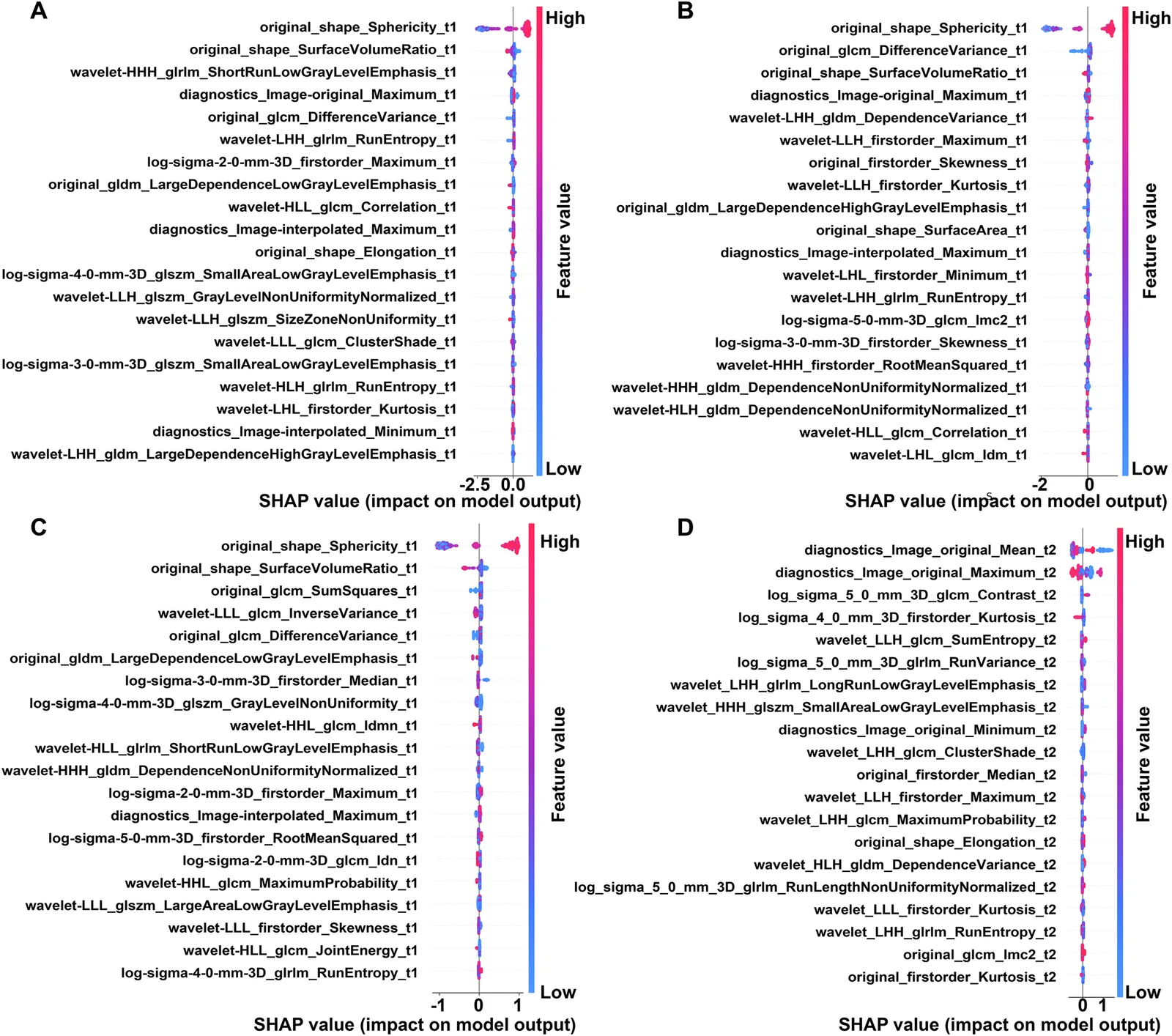
Global importance ranking diagram of SHAP scores for various prediction models. **A** EGFR, **B** 19Del, **C** L858R, and **D** 19Del or L858R.

To interpret the model’s decision-making process, SHapley Additive exPlanations (SHAP) were used to evaluate global and individual feature contributions. For Task 1, the most influential radiomic features were *original_shape_sphericity*, *original_shape_SurfaceVolumeRatio*, and *wavelet-HHH_GLRLM_ShortRunLowGreyLevelEmphasis* (Fig. 3A). For Task 2 (19Del), features such as *original_GLCM_DifferenceVariance* and *original_shape_SurfaceVolumeRatio* were particularly important (Fig. 3B). Task 3 (L858R) was largely driven by original_GLCM_SumSquares and shape features (Fig. 3C), while Task 4 was associated primarily with *diagnostics_image_original_Mean* and *diagnostics_image_original_Maximum* (Fig. 3D).

SHAP force plots (Supplementary Fig. 2) were generated for internal cohorts, highlighting how radiomic features contributed to individual predictions. In high-risk cases, SHAP values for EGFR, 19Del, and L858R mutations reached 0.15, 1.21, and 1.04, respectively. Conversely, in low-risk cases, SHAP values were negative (e.g., −2.86 for EGFR mutation), reflecting reduced mutation probability. Complementary analyses using Local Interpretable Model-Agnostic Explanations (LIME) further illustrated local feature contributions at the individual level (Supplementary Figs. 4 and 5). Red and blue bars indicated positive or negative contributions. While SHAP force plots illustrated patient-specific feature contributions, we observed that the locally important features identified by SHAP and LIME were not entirely identical, aside from consistently shared key features such as original_shape_Sphericity_t1. For instance, in a representative case predicted as EGFR-mutant with a probability of 1.00 (Supplementary Fig. 5A), LIME identified original_shape_Sphericity_t1 as the strongest positive contributor (weight = 0.26), consistent with its top global SHAP ranking. However, other features such as original_shape_SurfaceVolumeRatio_t1 (weight = −0.06) and log-sigma-2-0-mm-3D_glrlm_LongRunEmphasis_t1 (weight = −0.03) showed negative contributions at the individual level, despite being ranked as important features in the global SHAP analysis.

### Radiogenomic analysis

**Fig. 4:**
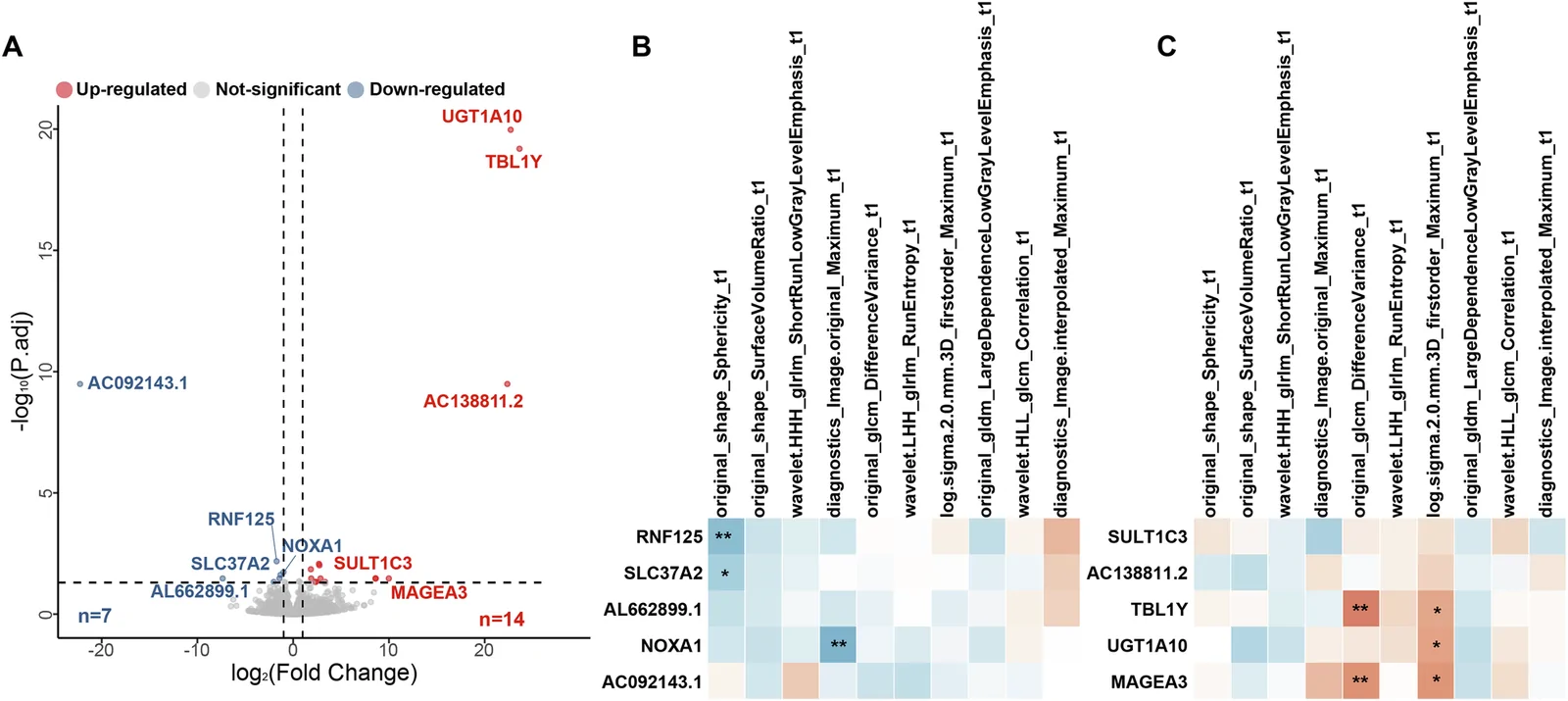
Transcriptomic interpretation of radiomics features associated with EGFR mutation status. **A** Differential gene expression analysis between the EGFR mutation and Non EGFR groups. **B** Correlation between the five most significantly downregulated genes in the EGFR mutation group and the model’s radiomics features. **C** Correlation between the five most significantly upregulated genes in the EGFR mutation group and the model’s radiomics features.

To explore the biological basis of radiomic phenotypes, we performed a radiogenomic analysis using RNA sequencing data from 38 patients who had paired MRI scans. Differential gene expression analysis between EGFR-mutant and wild-type groups identified 310 significantly altered genes (adjusted *P* < 0.05, |log₂FC| > 1), including upregulation of UGT1A10, TBL1Y, MAGEA3, and downregulation of RNF125, SLC37A2 (Fig. 4A).

Correlation analysis between gene expression and radiomic features revealed that TBL1Y and MAGEA3 were significantly associated with *original_GLCM_DifferenceVariance* (Task 2), while RNF125 and SLC37A2 negatively correlated with *original_shape_sphericity*, a top-ranked feature in Tasks 1–3 (Fig. 4B, C). Following Benjamini–Hochberg False Discovery Rate (FDR) correction for multiple testing, TBL1Y and MAGEA3 remained significantly associated with original_GLCM_DifferenceVariance in Task 2 (adjusted *P* = 0.002 and *P* = 0.009, respectively). RNF125 and SLC37A2 also showed robust negative correlations with original_shape_sphericity (adjusted *P* = 0.009 and *P* = 0.04, respectively).

Patients were further stratified based on *original_shape_sphericity* values into high- and low-expression groups (Supplementary Fig. 3A). EGFR mutations were significantly more prevalent in the high-expression group. Differential gene analysis between these two groups revealed 42 upregulated and 268 downregulated genes (Supplementary Fig. 3B). Key upregulated genes included NLRP2, SPNS2, PLCG2, BLNK, and PRIM2, while downregulated genes included SOHLH1, GDF1, GABRA5, and DUSP13 (Supplementary Fig. 3C, D).

**Fig. 5:**
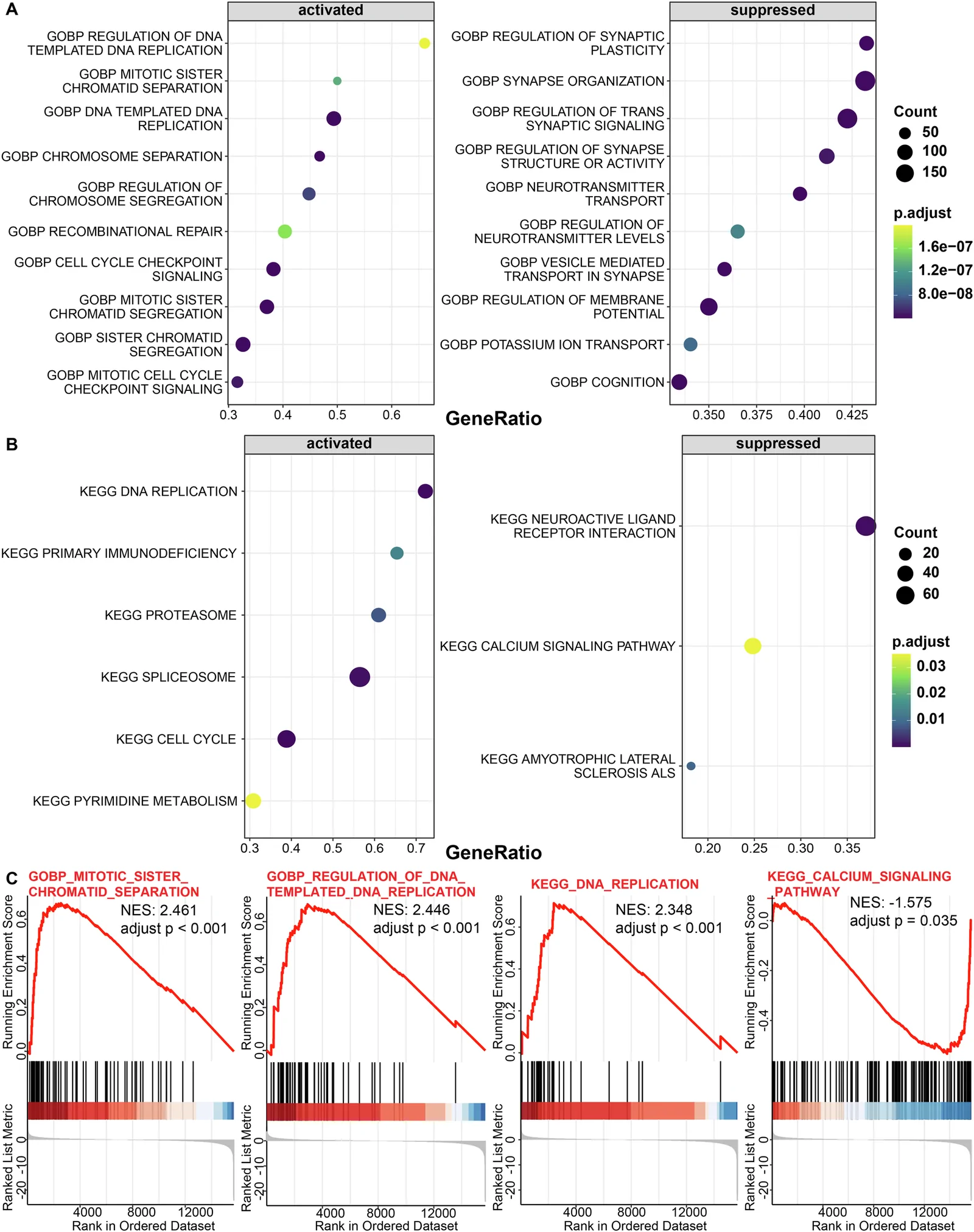
Enrichment analysis in radiogenomics includes. **A** GO enrichment analysis of 310 differentially expressed genes, displayed in a bubble plot of enriched GO terms; **B** a KEGG enrichment analysis of 310 differentially expressed genes, depicted in a bubble plot illustrating enriched KEGG pathways; **C** GSEA analyses.

Gene Ontology (GO) enrichment identified terms such as “DNA replication”, “sister chromatid segregation”, and “chromosome organization” (Fig. 5A). KEGG analysis highlighted pathways including “neuroactive ligand-receptor interaction”, “primary immunodeficiency”, and “calcium signaling” (Fig. 5B). GSEA further confirmed that the cell cycle, DNA replication, and mitotic chromatid segregation were among the most significantly enriched processes in the high-radiomic expression group (Fig. 5C).

## Discussion

MRI has shown promise as a non-invasive tool for inferring molecular subtypes in LUAD, particularly in identifying EGFR-mutant BMs. Yet, limited studies have effectively integrated radiomics with transcriptomic data to uncover the underlying biological mechanisms. This study addressed this gap by constructing a comprehensive radiogenomic model to predict EGFR mutation status in LUAD-BMs and elucidate the biological relevance of key radiomic features.

We developed four classification models targeting EGFR mutation types using a large multi-center cohort. Among various algorithms, the LightGBM combined with T1 sequences outperformed others in overall classification performance. The best model achieved an AUROC of 0.95 for EGFR status prediction, and over 0.90 for 19Del and L858R subtypes. Notably, the model was externally validated in a tissue-confirmed cohort and yielded an accuracy of 83%, confirming its robustness and clinical applicability.

Interestingly, unenhanced T1-weighted imaging alone achieved comparable, and in some cases superior, performance relative to multi-sequence feature fusion. This observation suggests that additional sequences do not necessarily provide incremental predictive value in this context. From a methodological perspective, this aligns with the principle of Occam’s razor.

From a biological and neuroradiological standpoint, one possible explanation is that unenhanced T1-weighted imaging primarily captures intrinsic tumor morphology, including structural boundaries and shape characteristics of the solid tumor component. Previous studies have reported that EGFR-mutant tumors may exhibit relatively well-circumscribed growth patterns, whereas wild-type tumors tend to show more heterogeneous or infiltrative margins^16^. It is therefore plausible that T1-derived radiomic features may partially reflect such morphological differences.

In contrast, T2-weighted and FLAIR sequences are highly sensitive to water content and peritumoral edema. While these features provide important information about the tumor microenvironment, they may also introduce variability related to non-specific factors such as inflammation and mass effect, potentially reducing their specificity for molecular subtyping. Similarly, contrast-enhanced T1-weighted imaging reflects blood–brain barrier disruption and vascular permeability, which may be influenced by both tumor biology and technical factors such as contrast timing^17^.

Interpretability was a key goal. We employed SHAP and LIME to deconstruct the decision-making process of our predictive models. Notably, the radiomic feature *original_shape_sphericity* consistently emerged as the most influential factor for distinguishing both overall EGFR mutation status and specific subtypes such as 19Del and L858R. This feature quantifies the similarity of the lesion to a perfect sphere, and higher values were associated with EGFR-mutant lesions. This aligns with previous research suggesting that wild-type EGFR LUAD tends to manifest as more irregular and heterogeneous tumors^18^.

Complementing these global findings, LIME analysis provides additional insight into individual-level model behavior. While SHAP captures global feature importance and overall contribution patterns, our results show that SHAP and LIME do not always identify identical feature contributions at the patient level, except for a subset of dominant features such as tumor shape descriptors. This discrepancy indicates that feature effects are context-dependent and can vary in both direction and magnitude across patients, likely reflecting nonlinear interactions among radiomic features. In this context, LIME approximates local decision boundaries and explicitly links feature contributions to predicted probabilities, enabling a more intuitive interpretation of high-confidence predictions. Rather than suggesting conflicting biological signals, these differences highlight the intrinsic complexity of radiomic phenotypes and underscore the limitations of relying on a single interpretability framework. Integrating global (SHAP) and local (SHAP and LIME) explanations, therefore, provides a more comprehensive and robust understanding of model behavior in heterogeneous tumors.

To explore the biological underpinnings of these imaging phenotypes, we conducted a multi-step radiogenomic analysis. Among the differentially expressed genes between EGFR-mutant and wild-type groups, RNF125 and SLC37A2 demonstrated strong negative correlations with *original_shape_sphericity*. RNF125 encodes a RING-finger ubiquitin ligase implicated in the degradation of immune and oncogenic proteins. Reduced RNF125 expression has been associated with enhanced tumor progression in melanoma and hepatocellular carcinoma, and our results suggest a potential similar role in LUAD, possibly through suppression of immune evasion pathways^19,20^. SLC37A2, a gene encoding a glucose-6-phosphate antiporter, is less well studied in the context of LUAD but is thought to regulate immune cell metabolism. Downregulation of SLC37A2 may contribute to immune escape and tumor progression, in part by altering the tumor microenvironment’s metabolic landscape^21,22^. Importantly, our results suggest that tumors with low expression of these genes exhibit more spherical, less infiltrative morphologies, providing a bridge between shape metrics and molecular aggression.

We further examined another radiomic marker, *original_GLCM_DifferenceVariance*, which measures texture heterogeneity by assessing intensity differences between neighboring voxels. High values denote greater heterogeneity, which often reflects increased tumor complexity. We found this feature to be strongly associated with the expression of TBL1Y and MAGEA3. TBL1Y, a Y-chromosome gene, is implicated in transcriptional co-repression and Wnt signaling, though its role in LUAD is unclear^23,24^. Our study is the first to suggest a potential link between TBL1Y and tumor radiomic heterogeneity. MAGEA3, a well-known cancer/testis antigen, has been studied extensively for its role in antigen presentation and is currently under clinical investigation as a target for cancer immunotherapy. The correlation of MAGEA3 with *GLCM_DifferenceVariance* implies that tumors with high heterogeneity may be more immunogenic and potentially more responsive to antigen-specific therapies. Particularly in 19Del -mutant BMs, where MAGEA3 expression is elevated, radiomics may serve as a surrogate marker for stratifying patients for vaccine-based or immunotherapeutic strategies^25–28^.

To explore shape-dependent biological mechanisms more broadly, we stratified patients based on *original_shape_sphericity* levels and conducted transcriptomic analyses. The high-expression group (more spherical lesions) had significantly more EGFR-mutant lesions and showed upregulation of genes such as PLCG2, BLNK, SPNS2, and PRIM2. Notably, PLCG2 is known to regulate intracellular calcium signaling and has been linked to enhanced metastasis in NSCLC through mitochondrial respiration pathways^29,30^. BLNK (B-cell linker protein) is typically associated with adaptive immune responses, and its expression in LUAD may reflect changes in tumor-infiltrating lymphocytes or antigen presentation capacity. In contrast, downregulated genes such as SOHLH1, GDF1, and GABRA5 have been linked to neural and endocrine differentiation, suggesting that more spherical tumors may adopt less differentiated, proliferative phenotypes.

Functional enrichment analyses further supported these observations. Gene Ontology and KEGG analyses revealed that differentially expressed genes were enriched in pathways related to DNA replication, mitotic spindle formation, and chromatid segregation, which are hallmarks of aggressive cell proliferation. Additionally, the ERBB signaling pathway, which includes EGFR, was activated in high-sphericity tumors^31,32^. GSEA reaffirmed these findings, demonstrating consistent upregulation of cell cycle-related signatures. These results suggest that the shape and texture of BMs, as quantified by radiomic features, reflect the proliferative and molecular aggressiveness of the tumor.

Our study also advances the interpretability and clinical trustworthiness of radiomics. By correlating image-derived features with molecular biomarkers and biological processes, we establish a stronger foundation for integrating radiomics into clinical decision-making. Radiogenomics not only enhances the predictive accuracy of models but also contextualizes their outputs within known tumor biology, helping clinicians to better understand, trust, and act upon model predictions.

Comparison with prior studies further underscores our contributions. Previous MRI-based models by Fan et al. and Cao et al. achieved AUROCs below or around 0.81, often relying on retrospective datasets without molecular validation^33,34^. In contrast, our study utilized a large retrospective training set, an independent external validation cohort, and a tissue-confirmed subgroup with paired sequencing data. The integration of SHAP/LIME interpretability tools and transcriptomic correlation represents a novel advancement that bridges quantitative imaging and biology.

However, several limitations should be acknowledged. First, although our data comprised three-dimensional MRI, we ultimately prioritized handcrafted radiomics over deep learning (DL) as the primary analytical framework. In the early phases of this study, we explored DL models with the anticipation of superior predictive performance. However, these efforts yielded suboptimal results. This observation aligns with our prior experience and a growing body of literature indicating that DL models often exhibit limited segmentation and classification accuracy for brain tumors, particularly in small- to moderate-sized datasets^2,11,35^. In such contexts, DL does not consistently surpass handcrafted radiomic features. Given our primary goals of precisely characterizing tumor parenchyma and establishing biologically interpretable radiogenomic associations, we opted for manually delineated ROIs, which offered greater interpretability.

Second, the spectrum of MRI sequences incorporated into our radiomics analysis was limited by the retrospective, multi-center design of the cohort. With increasing interest in BMs, additional conventional and functional sequences, such as fluid-attenuated inversion recovery (FLAIR), diffusion-weighted imaging (DWI), and advanced techniques like chemical exchange saturation transfer (CEST)/amide proton transfer (APT), have been shown to provide complementary biological and diagnostic insights^36,37^. However, in this study, pre-treatment FLAIR and DWI sequences were not uniformly available across all patients, nor were they acquired with consistent parameters across institutions. Consequently, reliable preprocessing, harmonization, and normalization could not be ensured, precluding their inclusion in the multi-sequence modeling. Furthermore, images were acquired using different scanner manufacturers and field strengths (1.5 T and 3.0 T). Although standard preprocessing (resampling, intensity normalization, and N4 correction) was rigorously applied, handcrafted features remain sensitive to scanner variability. In this study, we opted not to apply advanced batch-effect correction tools, such as ComBat, because the relatively small and imbalanced sample sizes within specific scanner subgroups could destabilize batch-effect parameter estimation, thereby introducing a risk of over-correcting and attenuating true biological variance. Consequently, the influence of inter-scanner heterogeneity cannot be entirely ruled out. Nevertheless, the robust predictive performance maintained in our completely independent external validation cohort suggests that our selected radiomic features possess an inherent resilience to this variability. Future large-scale, prospectively designed studies with balanced multi-center cohorts should incorporate robust harmonization strategies like ComBat to further mitigate batch effects and enhance model generalizability.

Third, while we investigated the biological significance of key radiomic features, notably original_shape_sphericity, through transcriptomic correlations and pathway enrichment analyses, these findings should be regarded as hypothesis-generating. Prior to interpreting their biological implications, we benchmarked these features against prior reports to confirm their association with EGFR-mutant LUAD BMs rather than representing nonspecific radiomic patterns^11,12^. Crucially, it must be acknowledged that our transcriptomic analyses were based on bulk RNA sequencing, which inherently captures not only tumor-intrinsic signals but also abundant components of the tumor microenvironment, such as reactive astrocytes, microglia, and infiltrating immune cells. Consequently, the observed associations suggest that macroscopic imaging phenotypes, like sphericity or texture heterogeneity, are integrated signatures. They likely reflect not only the intrinsic proliferation and invasion capabilities of the tumor cells but also the macroscopic physical compression, stromal remodeling, and complex immune infiltration within the surrounding brain tissue. Therefore, the mechanistic insights derived from bulk RNA sequencing and enrichment analyses require further validation through functional studies (in vitro or in vivo models) and more advanced approaches, such as single-cell or spatial transcriptomics. Accordingly, conclusions linking radiomic phenotypes to underlying molecular mechanisms should be viewed as exploratory rather than conclusive.

In conclusion, our study demonstrates that MRI-based radiomics can non-invasively predict EGFR mutation status in LUAD-BMs with high accuracy. The integration of SHAP-based model interpretability and transcriptomic correlation strengthens the biological plausibility of key imaging features. Notably, shape-based features such as *original_shape_sphericity* and texture features like *GLCM_DifferenceVariance* are not only statistically informative but also biologically meaningful, reflecting tumor proliferative and metabolic characteristics. By combining deep radiomics with transcriptomic profiling, our work paves the way for biologically-informed, interpretable AI models in neuro-oncology and precision medicine. Future studies should validate these findings in larger, prospective, multi-center cohorts and explore therapeutic implications of radiomic biomarkers for stratified EGFR-targeted therapies.

## Methods

### Study design and data collection

This retrospective, multicenter study was approved by the Institutional Review Board of Xiangya Hospital, Central South University (Approval No. 202210235), with informed consent waived due to its retrospective nature.

MRI and clinical data were obtained from three hospitals, forming two principal datasets along with an additional tissue-validated cohort:

Internal Cohort (*N* = 321 patients,·*n* = 1088 lesions): Retrospectively collected from Xiangya Hospital and Xiangya Bo’ai Rehabilitation Hospital between January 2016 and December 2022. This dataset included LUAD patients diagnosed with BMs, who were randomly assigned to a training cohort (70%) and a testing cohort (30%). To address class imbalance, the Synthetic Minority Over-sampling Technique (SMOTE) was applied exclusively to the training set after the train-test split. The test set remained completely independent and consisted solely of original, non-augmented samples. No synthetic samples were introduced into the validation or test cohorts, thereby preventing data leakage and ensuring an unbiased evaluation of model performance. All preprocessing steps, including feature selection and normalization, were also performed within the training set and subsequently applied to the test set to avoid information leakage.

External Validation Dataset (*N* = 47 patients, *n* = 121 lesions): Prospectively collected from the Second Affiliated Hospital of the University of South China between January 2024 and December 2024, this dataset served as an independent cohort for external validation of the radiomics model.

Tissue Validation Cohort (*N* = 53 patients, *n* = 94 lesions): Prospectively collected at Xiangya Hospital from January 2023 to September 2024. This cohort consisted of patients with EGFR mutation status confirmed directly from BMs, enabling biological validation of radiomic predictions to strengthen the reliability of the model.

### Inclusion and exclusion criteria

Eligible patients met the following criteria: (1) aged between 18 and 85 years; (2) confirmed EGFR mutation status via biopsy or surgical resection; and (3) availability of pre-treatment MRI sequences, including T1-weighted (T1WI), T2-weighted (T2WI), and contrast-enhanced T1-weighted (T1C) images. Patients were excluded if they had: (1) a history of other malignancies; (2) incomplete or missing MRI sequences; or (3) brain metastatic lesions smaller than 3 mm or with MRI matrix dimensions below 114 × 114 pixels.

### MRI acquisition parameters

The MRI scanning parameters were as follows:

At **Xiangya Hospital**, the median acquisition values for T1-weighted sequences were: repetition time (TR) = 477.6 ms and echo time (TE) = 13 ms. For T2-weighted sequences: TR = 4457.1 ms and TE = 110.2 ms. For contrast-enhanced T1-weighted (T1C) sequences: TR = 485.7 ms and TE = 13.1 ms.

At **Xiangya Bo’ai Rehabilitation Hospital**, three MRI systems were utilized: First, GE OPTIMA MR360 1.5 T: T1WI: TR = 1760 ms, TE = 24 ms; T2WI: TR = 6625 ms, TE = 115 ms. Second, GE Signa Pioneer 3.0 T: T1WI: TR = 2588 ms, TE = 8.5 ms; T2WI: TR = 4828 ms, TE = 101 ms. The last, GE Signa HDxt 3.0 T: T1WI: TR = 1987 ms, TE = 22.5 ms; T2WI: TR = 8602 ms, TE = 170 ms.

At the **Second Affiliated Hospital of the University of South China**, all patients underwent pre-treatment MRI using either 1.5 T or 3.0 T scanners. The imaging systems and corresponding parameters were: GE Signa Pioneer 3.0 T: T1WI: TR = 2588 ms, TE = 8.5 ms; T2WI: TR = 4828 ms, TE = 101 ms. GE Signa HDxt 3.0 T: T1WI: TR = 1987 ms, TE = 22.5 ms; T2WI: TR = 8602 ms, TE = 170 ms. GE OPTIMA MR360 1.5 T: T1WI: TR = 1760 ms, TE = 24 ms; T2WI: TR = 6625 ms, TE = 115 ms.

### Data preprocessing and radiomic feature extraction

Tumor ROIs were manually delineated using Insight Toolkit software (ITK-SNAP, version 3.8.0) on all axial slices across T1-weighted (T1), T2-weighted (T2), and contrast-enhanced T1-weighted (T1C) MRI sequences, ensuring the exclusion of cystic, hemorrhagic, and necrotic areas. Tumor boundaries were identified through visual consensus across the three sequences. In cases with multiple lesions larger than 3 mm, each lesion was individually delineated. All delineations were independently performed by two radiology residents (F.D. and X.C., each with over three years of training) and reviewed by an experienced neuroradiologist (R.Z., >20 years of experience) to resolve discrepancies. All radiologists were blinded to EGFR mutation status.

Image preprocessing was conducted in Python 3.6 using Simple ITK^38^. All MRI scans underwent gradient non-linearity correction, intensity normalization, and bias field correction via the N4 algorithm to remove low-frequency intensity variations. Subsequently, we applied a voxel-level 3D registration approach to precisely align corresponding voxels across sequences, significantly enhancing spatial accuracy for subsequent feature extraction. Images were resampled to a uniform voxel size of 0.375 × 0.375 × 6.5 mm, standardizing spatial resolution across datasets. Specifically, linear interpolation was utilized for the image intensities, while nearest-neighbor interpolation was applied to the ROI masks to preserve label integrity and avoid boundary distortion during feature extraction.

Radiomic features were extracted using PyRadiomics^39^. A total of 3435 features, including shape-based features, first-order intensity features, and texture features (GLCM, GLRLM, GLSZM, GLDM, and NGTDM), as well as wavelet- and LoG-filtered features, were extracted from ROIs across T1, T2, and T1C sequences. Feature dimensionality was subsequently reduced through a three-stage feature engineering process: (1) correlation-based filtering using pixel-level Spearman correlation analysis to remove redundant features; (2) feature normalization (*Z*-score normalization) to standardize data scales across samples; and (3) the intersection of selected features from the above two methods, creating a refined and robust feature set for predictive modeling.

1 $${\rm{Spearman\; correlation\; formula}}:{r}_{s}=1-\frac{6\sum {d}_{i}^{2}}{n({n}^{2}-1)}$$

Where *r*_*s*_ is the Spearman correlation coefficient, *d*_*i*_ represents the difference between the ranks of the corresponding variables for the *i-th* sample, and *n* is the total number of observations.

2 $${\rm{Normalization\; formula}}:{\rm{z}}=\frac{x-\mu }{\delta }$$

Where *z* represents the normalized feature value (*Z*-score), *x* is the original extracted feature value, μ is the mean of the feature values across the cohort, and δ is the standard deviation of the feature values.

### Model construction, validation, and interpretability

We defined four specific predictive tasks based on EGFR mutation types:

Task 1: Predict the presence or absence of any EGFR mutation.

Task 2: Identify patients specifically harboring the exon 19 deletion (19Del).

Task 3: Identify patients specifically harboring the exon 21 L858R mutation.

Task 4: Identify patients with either 19Del or L858R mutations.

To optimize model performance across these tasks, we implemented an Adaptive Optimization Training Pipeline (AOTP). AOTP dynamically adjusted hyperparameters, including learning rate, regularization strength, and kernel functions, in real time based on continuously monitored validation accuracy, facilitating efficient convergence, minimizing local minima entrapment, and enhancing model generalization. Additionally, AOTP incorporated adaptive early stopping, which terminated training when the moving average of validation performance plateaued, preventing overfitting. Eight classification algorithms, logistic regression (LR), support vector machine (SVM), decision tree (DT), random forest (RF), gradient boosting machine (GBM), multilayer perceptron (MLP), extreme gradient boosting (XGBoost), and Light Gradient Boosting Machine (LightGBM), were evaluated. Two distinct model fusion methods were employed to enhance predictive robustness:

Direct feature fusion: Imaging features from T1, T2, and T1C modalities were directly integrated into predictive models. For pairs of features identified as highly correlated (Spearman correlation coefficient > 0.9), a tie-breaking criterion was applied: the feature demonstrating a higher univariate Area Under the Curve (AUC) for the target outcome was retained, while the redundant feature was excluded.

Model stacking: Predictions from individual classifiers served as inputs to a logistic regression meta-classifier to derive final ensemble predictions.

Key AOTP parameters included the decay factor (0.1–0.5), progressively adjusting the learning rate; the importance factor (1.05–1.2), dynamically weighting critical features based on feature importance; and adaptive early stopping, utilizing a minimal improvement threshold (ε) and patience threshold (P epochs) to prevent overfitting.

Model performance was quantified by ROC curves and the AUC, with statistical differences assessed via the DeLong test (*p < 0.05, **p < 0.01, ***p < 0.001).

To interpret model predictions, SHAP provided global feature importance analysis, while LIME illustrated feature contributions on a local, case-by-case basis, clarifying the decision-making process behind EGFR mutation predictions.

### Radiogenomic analysis

Paired RNA sequencing data from 38 patients with matched MRI scans were available for radiogenomic analysis^40^. These patients were selected from the tissue validation cohort.

Radiogenomic analysis was conducted in two major steps: (1) constructing a radiogenomic correlation map linking imaging phenotypes with transcriptomic profiles, and (2) identifying candidate biological pathways and genes underlying radiomic signatures associated with EGFR mutation prediction.

Differential gene expression analysis was performed between EGFR-mutant and wild-type groups using the DESeq2 package in R. Significantly differentially expressed genes were defined by an adjusted *P*-value < 0.05 and |log₂ fold change | > 1. The top ten most differentially expressed genes (five upregulated, five downregulated) were analyzed for correlation with the top-ranked radiomic features identified in the EGFR classification model. Pearson correlation coefficients were calculated between gene expression values and radiomic feature scores. To account for multiple comparisons, the resulting *P*-values from the correlation analyses were adjusted using the Benjamini–Hochberg False Discovery Rate (FDR) method. Only correlations with an FDR-adjusted *P*-value < 0.05 were considered statistically significant and visualized in the radiogenomic heatmap.

To further investigate the molecular significance of key radiomic phenotypes (e.g., original_shape_sphericity), patients were stratified into high- and low-score groups based on optimal cutoff values derived from ROC curve analysis. Differential gene expression analysis between these groups revealed 310 significantly altered genes (42 upregulated, 268 downregulated), visualized using volcano plots and heatmaps. Pathway enrichment analysis was subsequently conducted to biologically annotate these gene sets. Gene Ontology (GO), Kyoto Encyclopedia of Genes and Genomes (KEGG), and Gene Set Enrichment Analysis (GSEA) were performed using clusterProfiler in R, with adjusted *P*-values < 0.05 considered significant. In addition, GeneMANIA was employed to construct a functional interaction network of the top 20 hub genes (10 upregulated and 10 downregulated), providing insight into the regulatory context of radiomic features.

## Supplementary information

Supplementary Files

## Data Availability

Data are available upon reasonable request to zhourr@csu.edu.cn. The raw sequence data reported in this paper have been deposited in the Genome Sequence Archive (Genomics, Proteomics & Bioinformatics 2021) in National Genomics Data Center (Nucleic Acids Res 2022), China National Center for Bioinformation / Beijing Institute of Genomics, Chinese Academy of Sciences (GSA-Human: HRA003286) that are publicly accessible at https://ngdc.cncb.ac.cn/gsa-human.
