# Supplementary material for: Radiogenomic modeling of EGFR mutation status in brain metastases from lung adenocarcinoma: a multicenter study with biological interpretability
Source: NPJ Digit Med. 2026 Jul 6;9:822. doi: 10.1038/s41746-026-02931-9 (PMC13720021; doi:10.1038/s41746-026-02931-9)
Supplement: Supplementary file 1 [file 41746_2026_2931_MOESM1_ESM.pdf]

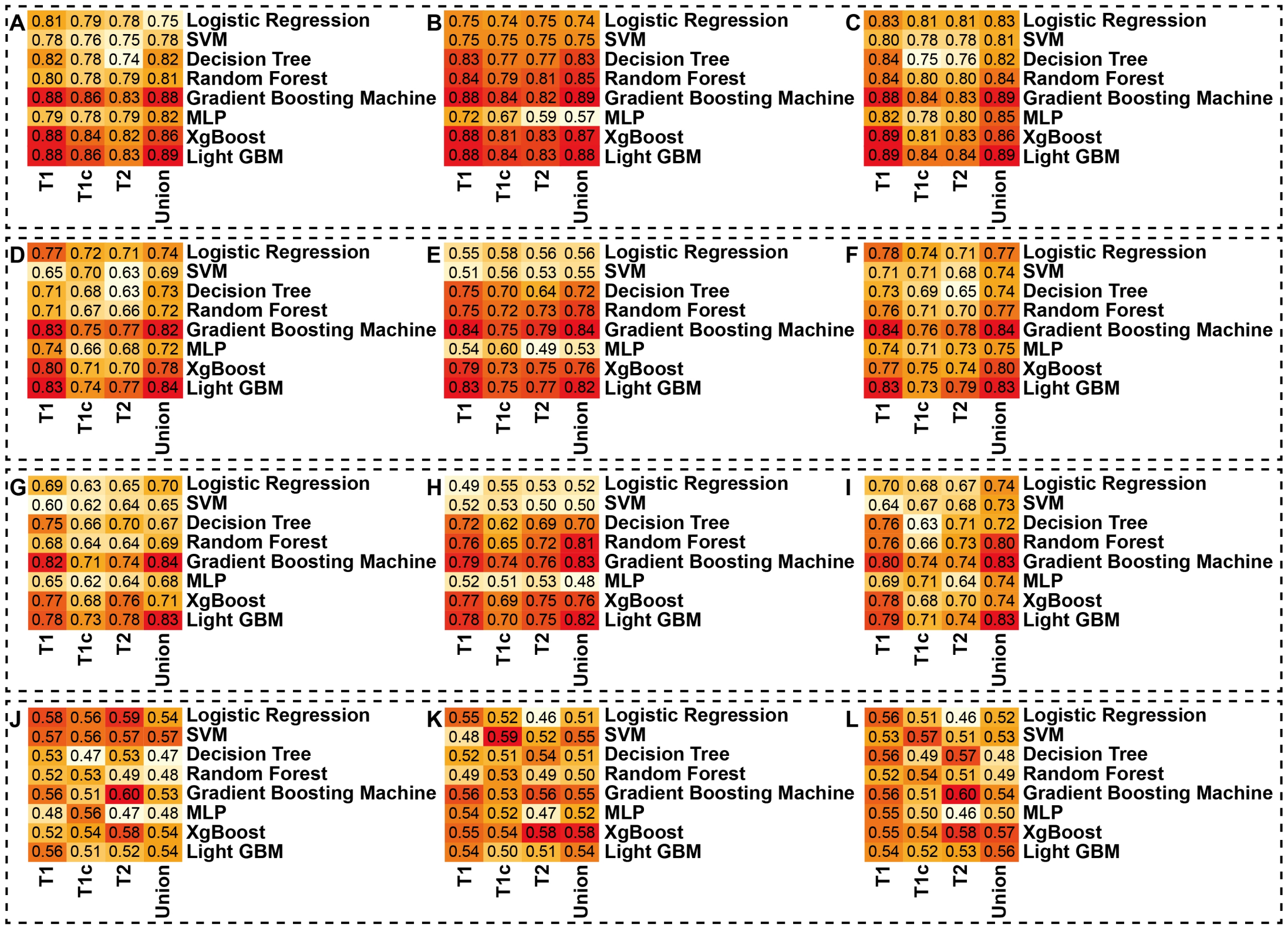

Supplementary Figure 1: Heatmaps of models' AUROC for selecting the optimal performance algorithm. Figures A, B, and C depict the predictions of EGFR mutations. Figures D, E, and F illustrate the predictions of Del19 mutations, while Figures G, H, and I predict the occurrence of L858R mutations. Figures J, K, and L predict either Del19 or L858R mutations. Specifically, Figures A, D, G, and J exclusively employ correlation-based feature filtering. Figures B, E, H, and K utilize only Z-score normalization. Figures C, F, I, and L represent feature engineering models that implement both correlation filtering and normalization methods. In summary, each row corresponds to a different classification task, and each column represents various feature processing methodologies.

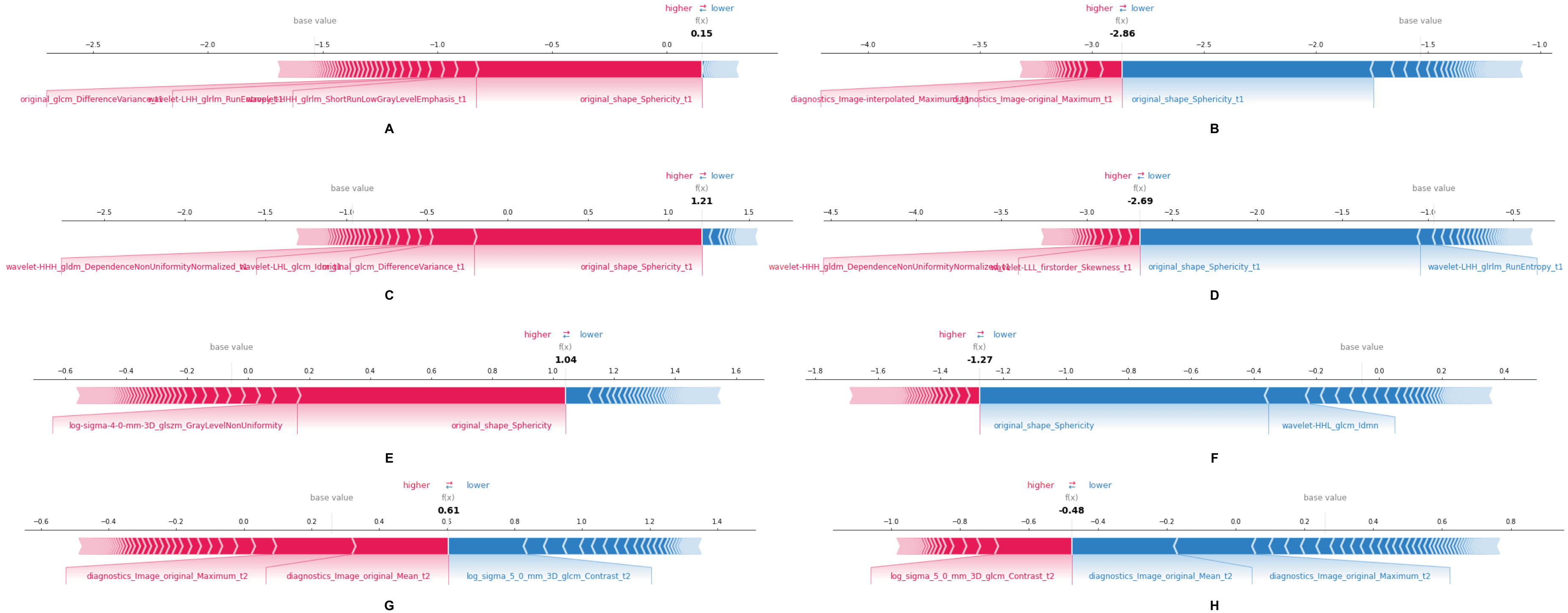

Supplementary Figure 2: Interpretability force plots of SHAP value predictions for individual patients. Panels (A) and (B) show overall EGFR mutation prediction for a patient with an EGFR mutation and a wild-type patient, respectively. Panels (C) and (D) illustrate Del19 mutation predictions for a Del19-mutant patient and a non-Del19 patient. Panels (E) and (F) depict L858R mutation predictions for an L858R-mutant patient and a non-L858R patient. Panels (G) and (H) represent predictions differentiating between Del19 and L858R mutations for respective patients.

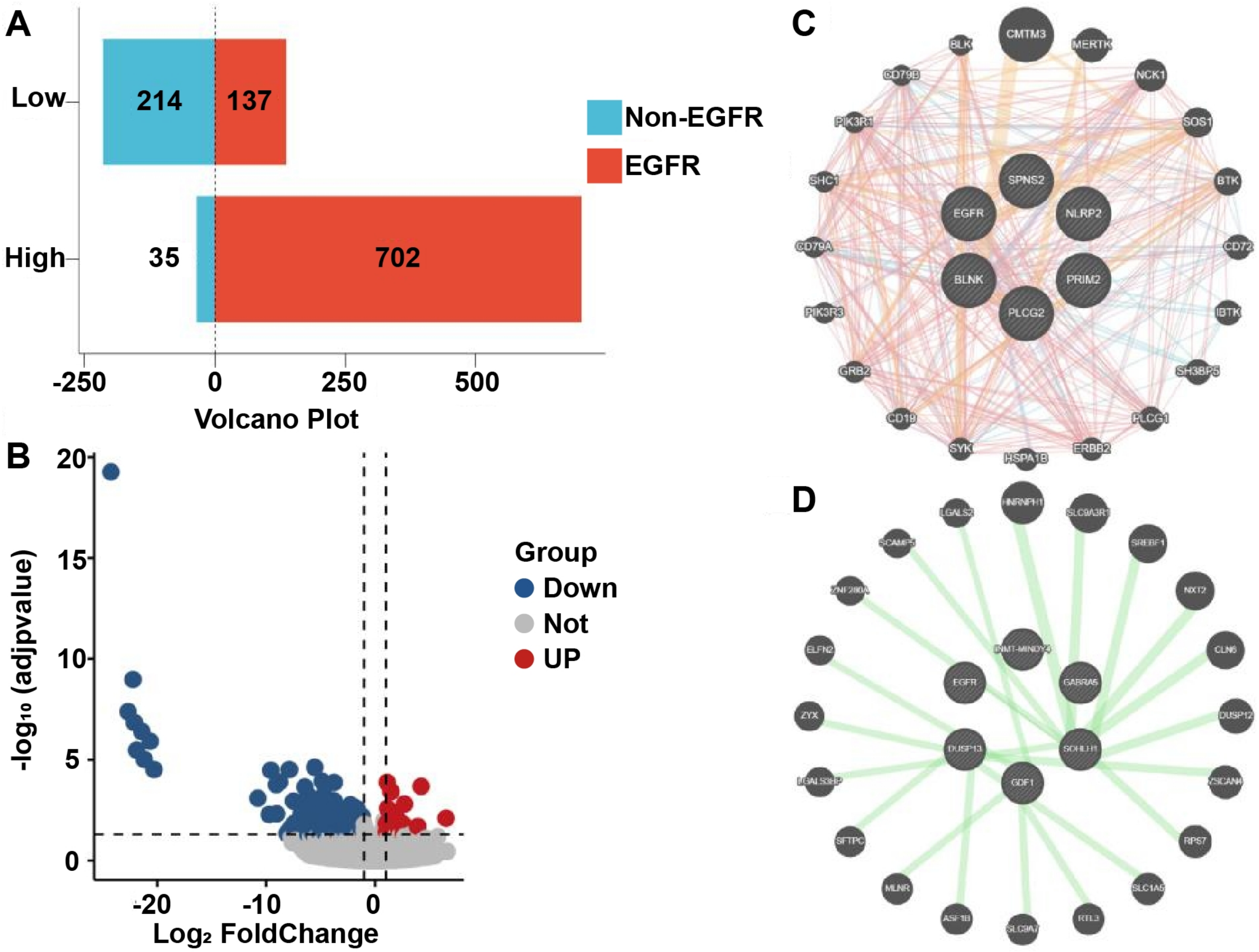

Supplementary Figure 3: Biological interpretability of shape radiomic features. (A) Comparison of the number of EGFR-mutant lesions in the high vs. low *Original_shape_sphericity* expression groups. (B) Differential gene expression analysis between the high and low *Original_shape_sphericity* expression groups. (C) Functional interaction network between the top 5 upregulated genes and EGFR. (D) Functional interaction network between the top 5 downregulated genes and EGFR.

A

Feature	Value
original_shape_Sphericity_t1	0.89
original_shape_SurfaceVolumeRatio_t1	0.96
log-sigma-2-0-mm-3D_ glrlm_LongRunEmphasis_t1	1.27
log-sigma-5-0-mm-3D_ glszm_SmallAreLowGrayLevelEmphasis_t1	0.20
wavelet-HHH-glszm_SmallAreaEmphasis_t1	0.92

B

Feature	Value
original_shape_Sphericity_t1	-2.16
original_shape_SurfaceVolumeRatio_t1	1.74
diagnostics_Image-original_Maximum_t1	0.85
wavelet-LLH_glszm_ SizeZoneNonUniformity_t1	0.20
wavelet-LHL_glcm_DifferenceAverage_t1	0.92

C

Feature	Value
original_shape_Sphericity_t1	-0.60
log-sigma-3-0-mm-3D_firstorder_Skewness_t1	-1.57
wavelet-HHH-glcm_ClusterTendency_t1	-0.45
wavelet-HLH_gldm_ GrayLevelNonUniformity_t1	-0.01
wavelet-HLL_glszm_ LargeAreaLowGrayLevelEmphasis_t1	-0.10

D

Feature	Value
diagnostics_Image_original_Mean_t2	768.04
log-sigma-4-0-mm-3D_firstorder_Kurtosis_t2	2.93
wavelet-LLL-firstorder_Kurtosis_t2	3.94
log_sigma_3_0_mm_ 3D_glcm_ClusterShade_t2	-1400.88
log_sigma_5_0_mm_ 3D_glcm_Contrast_t2	13.34

Supplementary Figure 4: Local feature contributions to the model's predictions derived from LIME analysis, distinguishing between the absence of mutation (Class 0) and the presence of the target mutation (Class 1). Panels (A) through (D) correspond to different mutation classification tasks: (A) overall EGFR, (B) Del19, (C) L858R, and (D) Del19 or L858R. Features are ranked vertically based on their impact on the final prediction. Each feature is represented by a bar, where the length signifies the strength of its local contribution. Blue bars represent features that drive the prediction towards Class 0 (wild-type/negative), while red bars represent those that push the prediction towards Class 1 (mutant/positive).

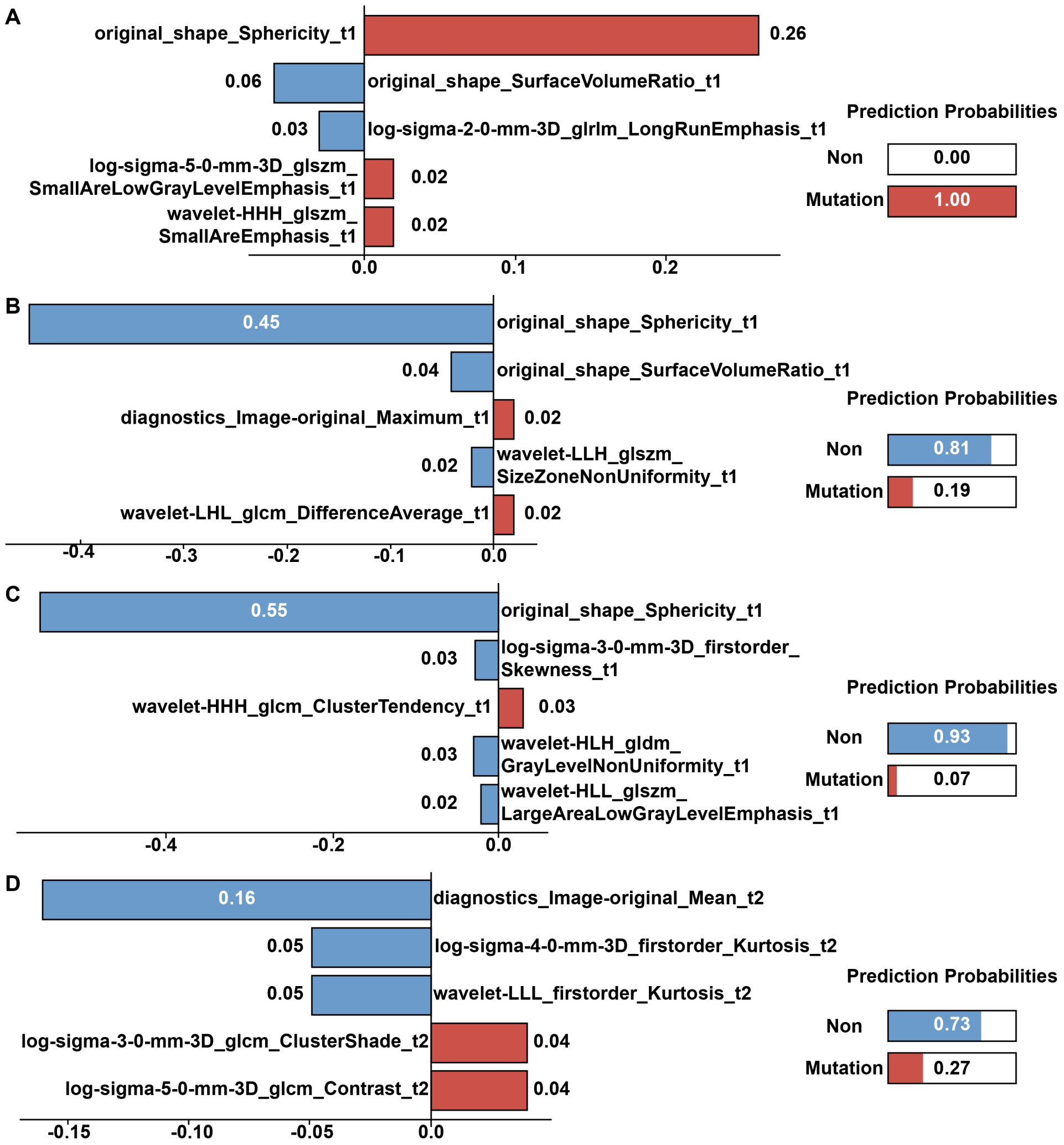

Supplementary Figure 5: Example outputs of Local Interpretable Model-agnostic Explanations (LIME) for individual patient predictions across four tasks: (A) overall EGFR mutation, (B) Del19 mutation, (C) L858R mutation, and (D) either Del19 or L858R mutation.

Supplementary Table1. Extraction methods and feature quantification of Radiomics features.

Feature class	Features	Feature class	Features
Firstorder	10Percentile	Gldm	DependenceEntropy
	90Percentile		DependenceNonUniformity
	Energy		DependenceNonUniformityNormalized
	Entropy		DependenceVariance
	InterquartileRange		GrayLevelNonUniformity
	Kurtosis		GrayLevelVariance
	Maximum		HighGrayLevelEmphasis
	MeanAbsoluteDeviation		LargeDependenceEmphasis
	Mean		LargeDependenceHighGrayLevelEmphasis
	Median		LargeDependenceLowGrayLevelEmphasis
	Minimum		LowGrayLevelEmphasis
	Range		SmallDependenceEmphasis
	RobustMeanAbsoluteDeviation		SmallDependenceHighGrayLevelEmphasis
	RootMeanSquared		SmallDependenceLowGrayLevelEmphasis
	Skewness	Glrlm	GrayLevelNonUniformity
	TotalEnergy		GrayLevelNonUniformityNormalized
	Uniformity		GrayLevelVariance
	Variance		HighGrayLevelRunEmphasis
Glszm	GrayLevelNonUniformity		LongRunEmphasis
	GrayLevelNonUniformityNormalized		LongRunHighGrayLevelEmphasis
	GrayLevelVariance		LongRunLowGrayLevelEmphasis
	HighGrayLevelZoneEmphasis		LowGrayLevelRunEmphasis
	LargeAreaEmphasis		RunEntropy

	LargeAreaHighGrayLevelEmphasis		RunLengthNonUniformity
	LargeAreaLowGrayLevelEmphasis		RunLengthNonUniformityNormalized
	LowGrayLevelZoneEmphasis		RunPercentage
	SizeZoneNonUniformity		RunVariance
	SizeZoneNonUniformityNormalized		ShortRunEmphasis
	SmallAreaEmphasis		ShortRunHighGrayLevelEmphasis
	SmallAreaHighGrayLevelEmphasis		ShortRunLowGrayLevelEmphasis
	SmallAreaLowGrayLevelEmphasis	Glcm	Autocorrelation
	ZoneEntropy		ClusterProminence
	ZonePercentage		ClusterShade
	ZoneVariance		ClusterTendency
	Busyness		Contrast
	Coarseness		Correlation
Ngtdm	Complexity		DifferenceAverage
	Contrast		DifferenceEntropy
	Strength		DifferenceVariance
	Elongation		Id
	Flatness		Idm
	LeastAxisLength		Idmn
	MajorAxisLength		Idn
Shape	Maximum2DDiameterColumn		Imc1/2
	Maximum2DDiameterRow		InverseVariance
	Maximum2DDiameterSlice		JointAverage
	Maximum3DDiameter		JointEnergy
	MeshVolume		JointEntropy
	MinorAxisLength		MCC

Sphericity	MaximumProbability
SurfaceArea	SumAverage
SurfaceVolumeRatio	SumEntropy
VoxelVolume	SumSquares

Supplementary Table 2: Pairwise statistical comparison of model performance across four tasks.

Task 1: Predict the presence or absence of any EGFR mutation

Model1	Model2	Delong Statistic	P Value
LogisticRegression	SVC		
LogisticRegression	DecisionTreeClassifier	-15.80461551	0
LogisticRegression	RandomForestClassifier	-12.35493895	0
LogisticRegression	GradientBoostingClassifier	-17.52726188	0
LogisticRegression	MLPClassifier		
LogisticRegression	XGBClassifier	-16.46425887	0
LogisticRegression	LGBMClassifier	-17.39518192	0
SVC	DecisionTreeClassifier	-15.89450426	0
SVC	RandomForestClassifier	-11.86972523	0
SVC	GradientBoostingClassifier	-17.54600746	0
SVC	MLPClassifier		
SVC	XGBClassifier	-16.12864017	0
SVC	LGBMClassifier	-17.06502306	0
DecisionTreeClassifier	RandomForestClassifier	5.920835569	3.2031E-09
DecisionTreeClassifier	GradientBoostingClassifier	-6.472957537	9.61029E-11
DecisionTreeClassifier	MLPClassifier	17.05996198	0
DecisionTreeClassifier	XGBClassifier	-3.447823021	0.000565124
DecisionTreeClassifier	LGBMClassifier	-4.776680309	1.78213E-06
RandomForestClassifier	GradientBoostingClassifier	-8.934819491	0
RandomForestClassifier	MLPClassifier	13.60353479	0
RandomForestClassifier	XGBClassifier	-6.756879567	1.40996E-11
RandomForestClassifier	LGBMClassifier	-9.013828213	0
GradientBoostingClassifier	MLPClassifier	18.3576316	0
GradientBoostingClassifier	XGBClassifier	4.487102884	7.21982E-06
GradientBoostingClassifier	LGBMClassifier	-0.115133488	0.908339317
MLPClassifier	XGBClassifier	-16.95612571	0
MLPClassifier	LGBMClassifier	-17.77286779	0
XGBClassifier	LGBMClassifier	-3.748394371	0.00017797

Task 2: Identify patients harboring the 19Del mutation

**Model1**	**Model2**	**Delong Statistic**	**P Value**
LogisticRegression	SVC	3096.282373	0
LogisticRegression	DecisionTreeClassifier	-1420.21721	0
LogisticRegression	RandomForestClassifier	-1388.888889	0
LogisticRegression	GradientBoostingClassifier	-3848.162072	0
LogisticRegression	MLPClassifier	3644.527987	0
LogisticRegression	XGBClassifier	-725.7727652	0
LogisticRegression	LGBMClassifier	-5612.99081	0
SVC	DecisionTreeClassifier	-4516.499582	0

SVC	RandomForestClassifier	-4485.171261	0
SVC	GradientBoostingClassifier	-6944.444444	0
SVC	MLPClassifier	548.245614	0
SVC	XGBClassifier	-3822.055138	0
SVC	LGBMClassifier	-8709.273183	0
DecisionTreeClassifier	RandomForestClassifier	31.3283208	0
DecisionTreeClassifier	GradientBoostingClassifier	-2427.944862	0
DecisionTreeClassifier	MLPClassifier	5064.745196	0
DecisionTreeClassifier	XGBClassifier	694.4444444	0
DecisionTreeClassifier	LGBMClassifier	-4192.773601	0
RandomForestClassifier	GradientBoostingClassifier	-2459.273183	0
RandomForestClassifier	MLPClassifier	5033.416876	0
RandomForestClassifier	XGBClassifier	663.1161236	0
RandomForestClassifier	LGBMClassifier	-4224.101921	0
GradientBoostingClassifier	MLPClassifier	7492.690058	0
GradientBoostingClassifier	XGBClassifier	3122.389307	0
GradientBoostingClassifier	LGBMClassifier	-1764.828739	0
MLPClassifier	XGBClassifier	-4370.300752	0
MLPClassifier	LGBMClassifier	-9257.518797	0
XGBClassifier	LGBMClassifier	-4887.218045	0

Task 3: Identify patients harboring the L858R mutation

Model1	Model2	Delong Statistic	P Value
LogisticRegression	SVC	2974.318658	0
LogisticRegression	DecisionTreeClassifier	-2528.825996	0
LogisticRegression	RandomForestClassifier	-3773.584906	0
LogisticRegression	GradientBoostingClassifier	-9748.427673	0
LogisticRegression	MLPClassifier	4664.570231	0
LogisticRegression	XGBClassifier	-8805.031447	0
LogisticRegression	LGBMClassifier	-11412.47379	0
SVC	DecisionTreeClassifier	-5503.144654	0
SVC	RandomForestClassifier	-6747.903564	0
SVC	GradientBoostingClassifier	-12722.74633	0
SVC	MLPClassifier	1690.251572	0
SVC	XGBClassifier	-11779.3501	0
SVC	LGBMClassifier	-14386.79245	0
DecisionTreeClassifier	RandomForestClassifier	-1244.75891	0
DecisionTreeClassifier	GradientBoostingClassifier	-7219.601677	0
DecisionTreeClassifier	MLPClassifier	7193.396226	0
DecisionTreeClassifier	XGBClassifier	-6276.205451	0
DecisionTreeClassifier	LGBMClassifier	-8883.647799	0
RandomForestClassifier	GradientBoostingClassifier	-5974.842767	0
RandomForestClassifier	MLPClassifier	8438.155136	0
RandomForestClassifier	XGBClassifier	-5031.446541	0

RandomForestClassifier	LGBMClassifier	-7638.888889	0
GradientBoostingClassifier	MLPClassifier	14412.9979	0
GradientBoostingClassifier	XGBClassifier	943.3962264	0
GradientBoostingClassifier	LGBMClassifier	-1664.046122	0
MLPClassifier	XGBClassifier	-13469.60168	0
MLPClassifier	LGBMClassifier	-16077.04403	0
XGBClassifier	LGBMClassifier	-2607.442348	0

Task 4: Identify patients with either 19Del or L858R mutations

Model1	Model2	Delong Statistic	P Value
LogisticRegression	SVC	inf	0
LogisticRegression	DecisionTreeClassifier	1.898904601	0.05757702
LogisticRegression	RandomForestClassifier	6.9478531	3.70881E-12
LogisticRegression	GradientBoostingClassifier	1881.109343	0
LogisticRegression	MLPClassifier	3.170421659	0.001522179
LogisticRegression	XGBClassifier	1.787712809	0.073822354
LogisticRegression	LGBMClassifier	12963.17345	0
SVC	DecisionTreeClassifier	-inf	0
SVC	RandomForestClassifier	-inf	0
SVC	GradientBoostingClassifier	-inf	0
SVC	MLPClassifier	inf	0
SVC	XGBClassifier	-inf	0
SVC	LGBMClassifier	inf	0
DecisionTreeClassifier	RandomForestClassifier	2523.30075	0
DecisionTreeClassifier	GradientBoostingClassifier	-466.0150034	0
DecisionTreeClassifier	MLPClassifier	6598.090475	0
DecisionTreeClassifier	XGBClassifier	-1176.403728	0
DecisionTreeClassifier	LGBMClassifier	10616.0491	0
RandomForestClassifier	GradientBoostingClassifier	-2989.315754	0
RandomForestClassifier	MLPClassifier	4074.789725	0
RandomForestClassifier	XGBClassifier	-3699.704478	0
RandomForestClassifier	LGBMClassifier	8092.748352	0
GradientBoostingClassifier	MLPClassifier	7.511119675	5.86198E-14
GradientBoostingClassifier	XGBClassifier	-710.3887247	0
GradientBoostingClassifier	LGBMClassifier	11082.06411	0
MLPClassifier	XGBClassifier	-19.38657078	0
MLPClassifier	LGBMClassifier	4017.958627	0
XGBClassifier	LGBMClassifier	11792.45283	0

Abbreviations: SVC = Support Vector Classifier; MLP = Multi-Layer Perceptron; XGB = eXtreme Gradient Boosting; LGBM = Light Gradient Boosting Machine; inf / -inf = positive or negative infinity (In the context of the DeLong test, an infinite test statistic occurs when the estimated variance of the difference between two ROC curves approaches zero, typically resulting from highly similar predictions or perfect separability in specific subsets, corresponding to a P-value of exactly 0).
